# Intellectual Impairment in School-Age Children Exposed to Manganese from Drinking Water

**DOI:** 10.1289/ehp.1002321

**Published:** 2010-09-20

**Authors:** Maryse F. Bouchard, Sébastien Sauvé, Benoit Barbeau, Melissa Legrand, Marie-Ève Brodeur, Thérèse Bouffard, Elyse Limoges, David C. Bellinger, Donna Mergler

**Affiliations:** 1 Centre for Interdisciplinary Studies in Biology, Health, Society and Environment (CINBIOSE), Université du Québec à Montréal, Montreal, Québec, Canada; 2 CHU Sainte-Justine Research Center, Université de Montréal, Montreal, Québec, Canada; 3 Department of Chemistry, Université de Montréal, Montreal, Québec, Canada; 4 Department of Civil, Geological and Mining Engineering, École Polytechnique de Montréal, Montreal, Québec, Canada; 5 Healthy Environments and Consumer Safety Branch, Health Canada, Ottawa, Ontario, Canada; 6 Department of Psychology, Université du Québec à Montréal, Montreal, Québec, Canada; 7 Department of Pedopsychiatry, Centre Hospitalier de l’Université Laval, Québec, Québec, Canada; 8 Department of Neurology, Boston Children’s Hospital, Harvard Medical School, Boston, Massachusetts, USA

**Keywords:** children, intellectual quotient, manganese, neurotoxicity, water

## Abstract

**Background:**

Manganese is an essential nutrient, but in excess it can be a potent neurotoxicant. Despite the common occurrence of manganese in groundwater, the risks associated with this source of exposure are largely unknown.

**Objectives:**

Our first aim was to assess the relations between exposure to manganese from drinking water and children’s intelligence quotient (IQ). Second, we examined the relations between manganese exposures from water consumption and from the diet with children’s hair manganese concentration.

**Methods:**

This cross-sectional study included 362 children 6–13 years of age living in communities supplied by groundwater. Manganese concentration was measured in home tap water (MnW) and children’s hair (MnH). We estimated manganese intake from water ingestion and the diet using a food frequency questionnaire and assessed IQ with the Wechsler Abbreviated Scale of Intelligence.

**Results:**

The median MnW in children’s home tap water was 34 μg/L (range, 1–2,700 μg/L). MnH increased with manganese intake from water consumption, but not with dietary manganese intake. Higher MnW and MnH were significantly associated with lower IQ scores. A 10-fold increase in MnW was associated with a decrease of 2.4 IQ points (95% confidence interval: −3.9 to −0.9; *p* < 0.01), adjusting for maternal intelligence, family income, and other potential confounders. There was a 6.2-point difference in IQ between children in the lowest and highest MnW quintiles. MnW was more strongly associated with Performance IQ than Verbal IQ.

**Conclusions:**

The findings of this cross-sectional study suggest that exposure to manganese at levels common in groundwater is associated with intellectual impairment in children.

Manganese is an essential nutrient involved in the metabolism of amino acids, proteins, and lipids, but in excess can be a potent neurotoxicant. Occupational and environmental exposure to airborne manganese has been associated with neurobehavioral deficits in adults and children (Riojas-Rodríguez et al. 2010; Zoni et al. 2007). In exposed workers, neurobehavioral deficits have been shown to correlate with manganese deposition in the brain observed by magnetic resonance imaging (Chang et al. 2009).

Manganese is commonly found in groundwater because of the weathering and leaching of manganese-bearing minerals and rocks into the aquifers; concentrations can vary by several orders of magnitude (Groschen et al. 2009). Because homeostatic mechanisms regulate manganese concentration in the organism, notably low absorption levels and a high rate of presystemic elimination by the liver (Roth 2006), it is generally believed that the oral route poses no significant toxic risk (Boyes 2010). Moreover, exposure to manganese from water consumption has been of little concern, because the intake of manganese from ingestion of water is small compared with that from foods, except for infants (Deveau 2010).

Few data are available on the risks from exposure to manganese from drinking water. One study in adults (Kondakis et al. 1989) and three studies in children (Bouchard et al. 2007; He et al. 1994; Wasserman et al. 2006) suggest that high manganese levels in water can be neurotoxic. In the Chinese province of Shanxi, 92 children 11–13 years of age, exposed to 240–350 μg manganese/L in water, had elevated hair manganese concentration (MnH), impaired manual dexterity and speed, short-term memory, and visual identification when compared with children from a control area (He et al. 1994). In Bangladesh, higher manganese concentration in water (MnW) was significantly associated with lower intelligence quotient (IQ) in 142 children 10 years of age; the mean MnW was 800 μg/L (Wasserman et al. 2006). In Quebec (Canada), our pilot study on 46 children 6–15 years of age showed that those exposed to higher MnW had significantly higher MnH, and the latter was associated with teacher-reported hyperactive and oppositional behaviors (Bouchard et al. 2007). Finally, two case reports show child manganese intoxication from water containing > 1,000 μg manganese/L, one presenting with attention and memory impairments (Woolf et al. 2002) and the other with neurologic symptoms including a repetitive stuttered speech, poor balance, coordination, and fine motor skills (Sahni et al. 2007).

Manganese concentration in drinking water is not regulated in the United States or Canada. Health-based guidelines for the maximum level of manganese in drinking water are set at 300 μg/L by the U.S. Environmental Protection Agency (EPA) (2004) and at 400 μg/L by the World Health Organization (WHO) (2008).

To date, no epidemiologic study has examined possible neurotoxic effects at manganese concentrations common in North American aquifers. In the present study, we assessed the relationship between exposure to manganese from drinking water and IQ of school-age children living in communities relying on groundwater. In addition, we examined the relations between MnH and estimated manganese intakes from water consumption and from the diet.

## Methods

### Study design and recruitment

We conducted this cross-sectional study in southern Quebec (Canada) between June 2007 and June 2009. Municipalities were considered as potential study sites if their aqueduct was supplied by groundwater. We selected eight municipalities to achieve a gradient of MnW. We explained the study to the principals and teachers of the elementary schools, who agreed to distribute recruitment letters to the children’s families. The response rate of the families was 60%, and the participation rate was 52%. Recruitment was restricted to children who had lived in the same house for > 3 months, to ensure continuous exposure to the same source of water for this minimum period of time; 362 children (age 6–13 years) participated in the study. The Human Research Ethics Board of the Université du Québec à Montréal approved the study protocol. We obtained signed informed consent from the parents and verbal assent from the child to participate in the study.

### Measurements of MnH

We collected a hair sample from the occiput of each child, cutting as close as possible to the root with surgical stainless steel scissors. We used the 2 cm closest to the scalp and washed samples to minimize external contamination, using the method described by Wright et al. (2006). In a test phase, we photographed hair strands with an electronic microscope and observed that the washing procedure effectively removed all particulates from the surface of the hair strand without compromising its structural integrity. We measured metals [manganese, lead (Pb), iron (Fe), arsenic (As), zinc (Zn), and copper (Cu)] by inductively coupled plasma-mass spectrometry (ICP-MS). Details of the analyses are in the Supplemental Material (doi:10.1289/ehp.1002321 via http://dx.doi.org/).

When manganese concentrations for certified hair material were outside of the designated concentrations, we excluded the measures from the analyses. Nine children who reported using hair dye in the preceding 5 months were also excluded, because hair dye could influence manganese hair content (Sky-Peck 1990). Children who reported use of hair dye had higher MnH compared with the others [geometric mean (GM), 1.1 μg/g, and 0.7 μg/g, respectively]. A total of 302 children were included in the analyses of MnH.

### Measurements of manganese and other elements in residential tap water

During the home visit, a parent responded to an interview-administered questionnaire about the source of the domestic tap water (private well/public well), residential history, and changes to domestic water treatments. We collected a water sample from the kitchen tap and also collected a second sample when there was a point-of-use filter (filter attached to the tap). We used the following procedure to standardize tap water sampling (van den Hoven and Slaats 2006): *a*) open the tap for 5 min, *b*) close and leave untouched for 30 min, and *c*) collect first draw. We added 0.15 mL nitric acid (50%) to the 50-mL water sample and stored samples at 4°C. We measured metals (manganese, Pb, Fe, As, Zn, and Cu) by ICP-MS. Calibration curves were run every 30 samples, along with field and laboratory blanks and quality controls (CRM TM-26.3; Environment Canada, Laboratory for Environmental Testing, Burlington, ON, Canada) every 15 samples.

For a subsample of participating families (*n =* 20), we sampled tap water on three occasions over a 1-year period to examine time-dependent variability.

### Estimation of manganese intake from the diet and water consumption

During the home visit, we orally administered a semiquantitative food frequency questionnaire to the parent and the child to assess manganese intake from the diet and water consumption. We used 3-dimensional models of portion size to obtain more precise estimates for all sites except the first site; thus, data for these participants (*n =* 16) were not included in the analyses on dietary intake. We estimated manganese intake from water consumption for direct water ingestion and for water incorporated in food preparations. We estimated water consumption from different sources—bottled, tap, tap filtered with a pitcher, and tap with an attached filter. For each water source, the amount consumed was multiplied by the measured or estimated concentration of manganese, yielding a total intake in micrograms per month. Further methodological details can be found in the Supplemental Material (doi:10.1289/ehp.1002321 via http://dx.doi.org/).

### Assessment of IQ and covariates

We used the Wechsler Abbreviated Scale of Intelligence (WASI) to assess general cognitive abilities (Wechsler 1999). This standardized test yields a Verbal IQ score (based on the subtests Vocabulary and Similarities), a Performance IQ score (Block Design and Matrix Reasoning), and a Full Scale IQ score. Throughout the study, three psychometricians administered the WASI, but all scoring was performed by the same person. We administered the WASI within 1 week of tap water sampling.

We collected information from the mother on factors that might confound the association between manganese exposure and cognitive abilities of the child, such as socioeconomic status indicators (i.e., maternal education, family income and structure), parity, and alcohol and tobacco consumption during pregnancy. We assessed maternal nonverbal intelligence with the Raven’s Progressive Matrices Test (Raven et al. 2003), home cognitive stimulation with a modified version of the Short-Form HOME (Home Observation for Measurement of the Environment) interview (Bradley et al. 2001), and maternal symptoms of depression with the Beck Depression Inventory-II (Beck et al. 1996). Data on family income were missing for four families, and the Raven score was missing for one mother; for missing data, we assigned the mean value of individuals with data.

### Statistical analysis

The distributions of manganese concentrations in hair and water, as well as manganese intakes, were considerably skewed. We thus employed log_10_ transformation to normalize residuals. Likewise, we log-transformed the concentrations of other elements measured in water. Manganese intakes from consumption of water and from the diet were divided by the weight of the child for use in the analyses (micrograms per kilogram per month). We used generalized estimating equations (GEE) to examine relationships between exposure to manganese and children’s IQ scores. GEE is an extension of generalized linear models for nonindependent data (Zeger and Liang 1986). These analyses were used to account for the community- and family-clustered data in our study. Some of the advantages of using GEE, instead of the more common approach of mixed models with random intercepts, include more efficient estimators of regression parameters and reasonably accurate standard errors [i.e., confidence intervals (CIs) with the correct coverage rates]. With GEE, the computational complexity is a function of the size of the largest cluster rather than of the number of clusters—an advantage and a source of reliable estimates when there are many small clusters (Hanley et al. 2003), such as in the present study (i.e., the 251 families). CIs were calculated with Wald statistics. An exchangeable working covariance matrix was used, with a robust estimator providing a consistent estimate of the covariance even when the working correlation matrix is misspecified.

Changes in IQ were examined in relation to four manganese exposure metrics reflecting different assumptions for exposure pathways and toxicokinetics: MnW, MnH, manganese intake from water consumption, and dietary manganese intake. The change in IQ (β) associated with a 10-fold increase in manganese exposure indicators was examined with adjustments for two sets of covariates. The first set of covariates (model A) was chosen based on the examination of directed acyclic graphs (Greenland and Brumback 2002) and included several socioeconomic indicators. Because manganese causes aesthetic problems, families who have the means might treat water domestically to remove it. The second set of covariates (model B) included the same variables as model A, as well as variables significantly associated with IQ or MnW, to reduce the unexplained variance, thus diminishing type 2 error (Bellinger 2007). We conducted sensitivity analyses on inclusion of additional covariates in the models. We used 0.05 as the threshold for statistical significance (two-sided tests). We examined residuals for normality and homoscedasticity and detected no problem.

## Results

### Descriptive statistics

This study included 362 children from 251 families. Most children (85%) had resided for > 12 months in their present home. The mean (± SD) age of the children was 9.3 ± 1.8 years; range, 6.2–13.4 years), and 99% of children were white. Seventy-eight percent of mothers had at least some college education (Table 1). Tap MnW ranged from 1 to 2,700 μg/L (Table 2), with an arithmetic mean of 98 μg/L and a GM of 20 μg/L. MnW from repeated sampling in the same residence over a period of 1 year had an intraclass correlation coefficient of 0.91.

MnW was not associated with socioeconomic or other family characteristics such as family income, family structure, home stimulation score, nonverbal maternal intelligence, or maternal education (Table 1). MnW was lower in houses with a private well (GM, 8 μg/L) than in those on a public well (GM, 55 μg/L). The concentration distribution for elements other than manganese in residential tap water is in the Supplemental Material (doi:10.1289/ehp.1002321 via http://dx.doi.org/). The Pearson correlation of MnW with other elements was 0.68 (Fe), 0.26 (Zn), 0.11 (Cu), 0.06 (As), and −0.02 (Pb).

### Estimated manganese intakes and children’s MnH

The median of estimated manganese intakes from direct consumption of water (1.6 μg/kg/month) was similar to the median of intakes from water incorporated into food preparations (1.9 μg/kg/month) (Table 2). The estimated dietary manganese intakes were much higher than the intakes from water consumption, with a median of 2,335 μg/kg/month (Table 2).

**Figure 1:**
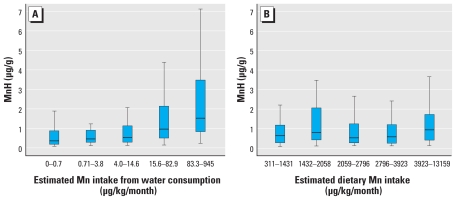
Distribution of MnH by quintiles of (*A*) estimated manganese intake from water consumption (*n =* 302), and (*B*) estimated manganese intake from the diet (*n =* 288). (Central bar: 50th percentile; lower and upper bounds of the rectangle: 25th and 75th percentiles; lower and upper tails: 5th and 95th percentiles. Observations outside the 95% CIs are not shown.)

Children’s MnH increased with MnW and estimated manganese intake from water consumption (Figure 1A), but not from the estimated dietary manganese intake (Figure 1B). In a multivariate model, MnH was significantly associated with manganese intake from water consumption (*p* < 0.001) but not with dietary intake (*p* = 0.76). In this multivariate model, there was no difference in MnH between boys and girls (*p* = 0.46; GM for both, 0.7 μg/g), and age was not associated with MnH (*p* = 0.88).

### Manganese exposure and children’s IQ

Estimated dietary manganese intake was not significantly associated with IQ scores in unadjusted or adjusted analyses (results not shown). Table 3 presents unadjusted and adjusted changes in IQ scores for a 10-fold increase in exposure level for three exposure indicators: MnW, estimated manganese intake from water consumption, and MnH. In unadjusted analyses, the three indicators were significantly associated with lower Full Scale IQ scores. Adjustment for covariates, with either model A or model B, did not considerably change the point estimates. Higher MnW was significantly associated with lower Full Scale IQ scores in model A [change in scores for a 10-fold increase in concentration (β) = −1.9 (95% CI, −3.1 to −0.7)] and model B [β = −2.4 (95% CI, −3.9 to −0.9)]. Higher MnW was also significantly associated with lower Performance IQ scores in model A [β = −2.3 (95% CI, −3.7 to −0.8)] and model B [β = −3.1 (95% CI, −4.9 to −1.3)]. Higher MnW was associated with lower Verbal IQ scores, significantly for model A but not for model B. (The point estimates were similar for both models but the 95% CI were larger for model B.) Higher estimated manganese intake from water consumption was significantly associated with lower Full Scale and Performance IQ, in both model A and model B. Sex-stratified analyses on MnW and Full Scale IQ resulted in higher point estimate for girls [using model B, β = −3.2 (95% CI, −5.0 to −1.5)] than for boys [β = −2.3 (95% CI, −4.8 to 0.2)], but the term for interaction with sex was not significant (*p* = 0.14).

When MnH was examined as the predictor of IQ in unadjusted analyses, it was significantly associated with lower Full Scale IQ scores but not Performance or Verbal IQ scores. In adjusted analyses, higher MnH was associated with lower Full Scale IQ scores, both in model A [β = −3.7 (95% CI, −6.5 to −0.8)] and in model B [β = −3.3 (95% CI, −6.1 to −0.5)]. MnH was also associated with lower Performance and Verbal IQ scores, although these relations did not reach statistical significance except for Verbal IQ in model A [β = −3.1 (95% CI, −5.9 to −0.3)]. Sex-stratified analyses resulted in higher point estimates for the association between MnH and Full Scale IQ for girls [β = −4.8 (95% CI, −8.1 to −1.6)] than for boys [β = −3.5 (95% CI, −8.7 to 1.6)], but the term for interaction with sex was not significant (*p* = 0.55).

**Figure 2:**
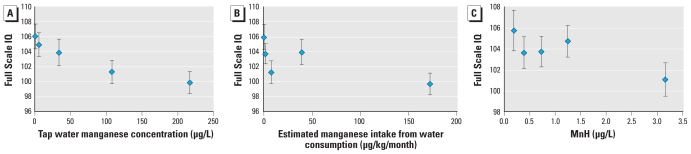
Mean Full Scale IQ (± SE), with respect to manganese exposure indicators, adjusted for covariates in model B. (*A*) IQ is plotted by median of tap water manganese concentration (μg/L) quintiles. The medians and ranges of MnW are as follows: 1st quintile (lowest), 1 (0–2); 2nd, 6 (3–19); 3rd, 34 (20–66); 4th, 112 (67–153); and 5th (highest), 216 (154–2,700). (*B*) IQ is plotted by median of manganese intake from water consumption (μg/kg/month) quintiles. The medians and ranges of manganese intakes are as follows: 1st quintile (lowest), 0.1 (0–0.7); 2nd, 1.6 (0.71–3.8), 3rd, 7.6 (4.0–14.6), 4th, 39.4 (15.6–82.9), and 5th (highest), 172 (83.3–945). (*C*) IQ is plotted by median of MnH (μg/g) quintiles. The medians and ranges of MnH are as follows: 1st quintile (lowest), 0.2 (0.1–0.3); 2nd, 0.4 (0.31–0.5); 3rd, 0.7 (0.51–0.9); 4th, 1.2 (0.91–1.9); and 5th (highest), 3.2 (1.91–20.7).

The shape of dose–response relations are shown in Figure 2. IQ scores decrease steadily with increasing MnW (Figure 2A). Children in the highest MnW quintile (median, 216 μg/L) scored 6.2 points below those in the lowest quintile (median, 1 μg/L). For estimated manganese intake from water ingestion (Figure 2B), children in the lowest quintile had the highest IQ scores, and those in the highest quintile had the lowest scores, but point estimates in the middle quintiles did not show a consistent pattern of increasing or decreasing trend. For MnH (Figure 2C), IQ scores decreased only slightly between children in the lowest quintile and the middles quintiles, and there was a steeper decrease for children in the highest quintile. A similar plot for dietary manganese intake showed no association, even in the higher range of intakes (data not shown).

We conducted sensitivity analyses on the inclusion of additional covariates in the models: birth weight of the child, rank of the child in the family, maternal smoking or alcohol consumption during pregnancy, maternal depressive symptoms, psychometrician, and concentration of Pb, As, Cu, and Zn in tap water. We did not retain these covariates, because they did not change point estimates by > 10% and were not significantly associated with IQ scores (*p* > 0.2).

## Discussion

The present study shows that children exposed to higher concentration of manganese in tap water had lower IQ scores. This finding was robust to adjustment for socioeconomic status indicators and other metals present in water. The association between MnW and IQ scores was strong, with a 6.2 Full Scale IQ point difference between the children exposed to water with 1 and 216 μg manganese/L (median of lowest and highest quintiles). Manganese intake from water ingestion, but not from the diet, was significantly associated with elevated manganese concentration in children’s hair. These findings suggest that manganese exposure from drinking water is metabolized differently than that from the diet and can lead to overload and subsequent neurotoxic effects expressed by intellectual impairments in children.

The communities included in the present study were chosen to ensure a gradient of manganese concentrations in drinking water. This selection was not random, and levels are not representative of the distribution of manganese concentration in wells used for human consumption in Canada or the United States. Nonetheless, the concentrations measured are not unusual in northeastern America. In New England, 45% of wells for public use have manganese concentrations > 30 μg/L (Groschen et al. 2009). Throughout the United States, approximately 5% of domestic household wells have concentrations > 300 μg/L (U.S. Geological Survey 2009). Elevated manganese in groundwater is common in several countries including Sweden (Ljung and Vahter 2007), Vietnam (Agusa et al. 2006), and Bangladesh (Wasserman et al. 2006).

The present findings are consistent with the two previous studies examining drinking water manganese-related cognitive deficits in children, albeit in the present study the mean manganese concentration was considerably lower than in the others: 100 μg/L versus approximately 300 μg/L (He et al. 1994) and 800 μg/L (Wasserman et al. 2006). Similar to our findings, Wasserman et al. (2006) observed a stronger association of water manganese level with Performance IQ than with Verbal IQ.

The different manganese exposure indicators showed consistent associations with lower IQ scores, although the shape of the dose–response curve differed by exposure indicators. Full Scale IQ scores decreased steadily with increasing MnW, but a discernable diminution in IQ was present only at higher concentrations of MnH. Interestingly, tap water manganese concentration was a better predictor of children’s IQ scores than the estimated intake from water ingestion, possibly because of error measurement in intakes. However, there may be pathways of exposure not captured by assessment of the ingested dose, such as inhalation of aerosols containing manganese ions in the shower (Elsner and Spangler 2005), although this hypothesis is debated (Aschner 2006). More studies are needed on manganese toxicokinetic and neurotoxicity to better understand these findings.

Our study does not address the mechanisms involved in manganese-related cognitive impairments, but an extensive body of literature shows perturbation of neurotransmitter activities (Olanow 2004), notably, disruption of the striatal dopaminergic system. Studies also reported data suggestive of perturbations of gamma-aminobutyric acid and serotonin (Dobson et al. 2004; Eriksson et al. 1987). Manganese effects can be persistent; adult mice exposed by gavage as juveniles had decreased striatal dopamine activity (Moreno et al. 2009). In nonhuman primates, chronic manganese exposure causes accumulation of the metal in the basal ganglia, white matter, and cortical structures, with signs of neuronal degeneration (Guilarte et al. 2006, 2008). Perturbations in the regulation of other metals in the brain could also be implicated in the cognitive impairment associated with manganese exposure (Fitsanakis et al. 2009).

In this study, dietary manganese intake was similar to the recommended dietary allowance of 1.5–1.9 mg/day for children 6–13 years of age (Food and Nutrition Board 2004). Manganese intake from ingestion of water was very small compared with the amount ingested from foods (by more than two orders of magnitude), yet only intake from water was significantly associated with MnH content. Previous studies have likewise reported a relation between the concentration of manganese in drinking water and hair (Agusa et al. 2006; Bouchard et al. 2007; He et al. 1994; Kondakis et al. 1989). This suggests that there might be differences in the regulation of manganese present in food and water. The chemical form of manganese, notably the valence state and solubility, might modify its toxicity, perhaps because of changes in toxicokinetic properties (Michalke et al. 2007). Moreover, manganese absorption is decreased in the digestive system with concurrent intake of dietary fiber, oxalic acids, tannins, and phytic acids (Gibson 1994).

Currently, no consensus has emerged as to the optimal biomarker of exposure to manganese (Smith et al. 2007). Even at very high MnW, no relation was observed between water manganese and blood manganese in children (Wasserman et al. 2006) or adults (Kondakis et al. 1989). Blood manganese can vary widely in the short term and thus might not reflect long-term exposure. For instance, in a case report of suspected manganese intoxication, three consecutive blood samples for plasma manganese on a single day showed large variability: 0.6, 2.2, and 2.4 μg/L (Sahni et al. 2007). In contrast, the manganese content in hair will reflect the metal uptake averaged over the duration of the follicle formation. The mechanism of manganese uptake into hair is not well understood, but its affinity for melanin, a protein present in hair, skin, and the central nervous system, could be involved (Lyden et al. 1984).

The strengths of our study include a larger sample size than in previous studies and thorough assessment of manganese exposure by ingestion, including from dietary sources. Repeated water sampling in the same house showed little variation in manganese concentration over the year, suggesting that one measure is representative of long-term exposure. The other metals present in tap water did not affect the association between manganese and IQ. We did not measure nonmetal water constituents; however, our findings are not likely to be explained by anthropogenic contaminants, because the observed manganese concentrations represent natural background levels associated with the bedrock geology and not human activities. There are no industrial sources of manganese emission in the study area, and since 2004, the gasoline additive methylcyclopentadienyl manganese tricarbonyl is no longer used in Canada (Finkelstein and Jerrett 2007).

Our study has limitations. As in any epidemiologic study, associations could be attributable to unmeasured confounders, but manganese concentration in tap water was completely dissociated from socioeconomic status, which diminishes the potential for confounding. Some level of exposure misclassification is expected, because we considered only manganese exposure from water consumed at home. However, nondifferential misclassification would most likely bias estimates of manganese associations with IQ toward the null. Despite measures taken to wash hair samples, residual external contamination cannot be ruled out completely. For instance, arsenic present in water has been shown to bind to the hair surface and was not removable by sample washing (Concha et al. 2006); it is not known whether this could also apply to manganese.

The inferences that can be drawn from the present findings are limited by its cross-sectional design. It is not known whether exposure during a critical developmental period is responsible for our observations. Most children in this study had been exposed to the current MnW for > 1 year; however, we did not attempt to assess exposure for more remote periods because retrospective data are often unreliable and this was not our initial objective. Studies employing a prospective design would provide a stronger basis for examining the influence of exposure duration and timing (i.e., critical developmental periods) on manganese neurotoxic effects. Notably, infants and young children could be at risk because of not fully developed homeostatic mechanisms, limiting absorption of ingested manganese (Ljung and Vahter 2007; Winder 2010).

## Conclusions

Manganese intake from water ingestion, but not from the diet, was significantly associated with elevated MnH, suggesting that homeostatic regulation of manganese does not prevent overload upon exposure from water. The findings from our study support the hypothesis that low-level, chronic exposure to manganese from drinking water is associated with significant intellectual impairments in children. These findings should be replicated in another population. Because of the common occurrence of this metal in drinking water and the observed effects at low MnW, we believe that national and international guidelines for safe manganese in water should be revisited.

## Figures and Tables

**Table 1:** Manganese concentrations in domestic tap water (μg/L) by characteristics of participant.

Characteristic	Frequency	Percent	MnW (GM)	*p-*Value^a^
Sex of child^b^				0.71

Male	168	46	19	
Female	194	54	21	

Child drinks tap water^b^				0.41

No	121	33	23	
Yes	241	67	19	

Maternal smoking during pregnancy^b^				0.13

No	265	73	18	
Yes	97	27	27	

Maternal alcohol consumption during pregnancy^b^				0.10

No	310	86	22	
Yes	52	14	13	

Home tap water source^c^				< 0.001

Private well	117	47	8	
Public well	134	53	55	

Family income^c^				0.28

≤ Can$50,000	106	42	27	
> Can$50,000	145	58	20	

Family structure^c^				0.87

Two biological parents	189	75	22	
One biological and one nonbiological parent	37	15	21	
Single parent	25	10	28	

Maternal education^c^				0.86

Less than high school	11	4	13	
High school diploma	44	18	24	
Some college	116	46	24	
Some university	80	32	21	

Nonverbal maternal intelligence (Raven)^c^				0.71

< 23	94	38	110	
23–25	94	38	84	
> 25	63	25	123	

Maternal depressive symptoms (Beck-II score)^c^				0.08

Normal range	206	82	24	
Mild symptoms	34	14	11	
Moderate or severe symptoms	11	4	40	

a Difference in MnW, from univariate general linear models.

b One measure per child (*n* = 362).

c One measure per family (*n* = 251).

**Table 2:** Distribution of concentrations for manganese in drinking water and children’s hair, as well as manganese intakes from water consumption and dietary sources.

			Percentile					
Manganese exposure indicators	*n*	Min	5th	25th	50th	75th	95th	Max
Manganese concentrations								

Tap water manganese (μg/L)	362	0.1	0.5	2.5	30.8	128	255	2,700
Hair manganese (μg/g)	302	0.1	0.2	0.3	0.7	1.6	4.7	21

Manganese intakes (μg/kg/month)								

From drinking water	362	0.0	0.0	0.0	1.6	22.9	160	566
From water used in food preparations	362	0.0	0.0	0.2	1.9	14.5	149	480
Total intake from water consumption	362	0.0	0.0	1.0	8.0	59.6	286	945
From dietary sources	346	311	840	1,632	2,335	3,487	6,418	13,159

Abbreviations: Max, maximum; Min, minimum.

**Table 3:** Unadjusted and adjusted changes in children’s IQ for a 10-fold increase in indicators of manganese exposure [β [95% CIs)].

Model	MnW (*n =* 362)	Manganese intake from water consumption (*n* = 362)	MnH (*n =* 302)
Unadjusted model			

Full Scale IQ	−2.1 (−3.5 to −0.8)**	−1.3 (−2.5 to −0.2)*	−3.2 (−6.2 to −0.2)*
Performance IQ	−2.4 (−4.0 to −0.7)**	−1.6 (−3.0 to −0.3)*	−2.5 (−5.7 to 0.8)
Verbal IQ	−1.4 (−2.6 to −0.2)*	−0.7 (−1.7 to 0.3)	−2.8 (−5.6 to 0.5)

Adjusted model A^a^			

Full Scale IQ	−1.9 (−3.1 to −0.7)**	−1.2 (−2.3 to −0.1)*	−3.7 (−6.5 to −0.8)*
Performance IQ	−2.3 (−3.7 to −0.8)**	−1.6 (−2.9 to −0.3)*	−3.0 (−6.1 to 0.1)
Verbal IQ	−1.5 (−2.6 to −0.3)*	−0.6 (−1.6 to 0.3)	−3.1 (−5.9 to −0.3)*

Adjusted model B^b^			

Full Scale IQ	−2.4 (−3.9 to −0.9)**	−1.2 (−2.3 to −0.1)*	−3.3 (−6.1 to −0.5)*
Performance IQ	−3.1 (−4.9 to −1.3)**	−1.9 (−3.3 to −0.4)*	−2.8 (−5.9 to 0.4)
Verbal IQ	−1.2 (−2.7 to 0.3)	−0.3 (−1.4 to 0.7)	−2.7 (−5.4 to 0.1)

a Adjusted for maternal education (less than high school/high school diploma/some college/some university) and nonverbal intelligence, family income, home stimulation score, and family structure (two biological parents/one biological and one nonbiological parent/single parent).

b Adjusted for same variables as above, and sex and age of child, IQ testing session (started at 0900/1300/1500), source of water (private well/public well), and Fe concentration in tap water.

* *p* < 0.05.

** *p* < 0.01.
